# Design of a Mobile and Electromagnetic Emissions-Compliant Brain Positron Emission Tomography (PET) Scanner

**DOI:** 10.3390/s25175344

**Published:** 2025-08-28

**Authors:** Cristian Fuentes, Marina Béguin, Volker Commichau, Judith Flock, Anthony J. Lomax, Shubhangi Makkar, Keegan McNamara, John O. Prior, Christian Ritzer, Carla Winterhalter, Günther Dissertori

**Affiliations:** 1 Institute for Particle Physics and Astrophysics, ETH Zurich, 8093 Zurich, Switzerland; 2 Center for Proton Therapy, PSI, 5232 Villigen, Switzerland; 3 Nuclear Medicine and Molecular Imaging Department, Lausanne University Hospital, 1011 Lausanne, Switzerland

**Keywords:** PET scanner, PET imaging, EMC, ICU, proton therapy

## Abstract

This paper presents the development of two mobile brain Positron Emission Tomography (PET) scanners under the PETITION project, designed for Intensive Care Units (ICUs) and Proton Beam Therapy (PBT) applications. The ICU scanner facilitates bedside imaging for critically ill patients, while the PBT scanner enables undisturbed proton beam irradiation during imaging. Key aspects of the hardware design, including modular detectors and electromagnetic interference considerations, are discussed along with preliminary performance evaluations. Operational testing, employing a ^22^Na source and a hot-rod phantom, was conducted to determine the timing resolution (548 ps), energy resolution (11.4%) and a qualitative spatial resolution (around 2.2 mm). Our study presents findings on the ICU PET scanner’s electromagnetic emissions measured in a controlled EMC testing facility, where all the emissions tests performed comply with the standard EN 60601-1-2 (radiated emissions 15 dB below regulatory limits in the frequency range of 30 MHz to 1 GHz).

## 1. Introduction

Positron Emission Tomography (PET) is a powerful molecular imaging technique that provides unique insights into in vivo physiological and metabolic processes, offering functional information that is complementary to the anatomical detail provided by modalities such as Computed Tomography (CT) or Magnetic Resonance Imaging (MRI). Its ability to quantify biological activity has made it an indispensable tool in both clinical diagnostics and biomedical research, particularly in the fields of neurology and oncology. However, the utility of conventional PET is fundamentally constrained by the large, stationary nature of commercially available PET scanners, which requires patients to be transported to dedicated imaging sites. This limitation presents a significant barrier in specialized clinical environments where patient mobility is compromised or where imaging must be integrated directly with therapeutic procedures.

Two such environments requiring mobile, high-performance PET technology are the Intensive care unit (ICU) and Proton Beam Therapy (PBT). Firstly, ICU patients, often too unstable to be moved, could greatly benefit from functional brain imaging to understand complex neurological conditions, such as delirium in deeply sedated patients [1]. Secondly, in PBT, there is a pressing need for in vivo verification of treatment delivery. In-beam PET imaging offers a non-invasive method for real-time proton range verification [2] and daily biological adaptation of treatment depending on the oxygen concentration in the tumor [3]. Both applications require mobile brain PET scanners that can perform similarly to full-body PET systems used in clinical settings.

Despite this clinical need, no commercially available mobile PET system fulfills our specific requirements. According to our review, while there are some systems documented in the literature [4,5,6,7,8], these models consistently fall short of the combined requirements for our target applications. Specifically, they often lack an aperture for proton beam irradiation, making them unsuitable for in-beam PBT applications. Furthermore, they either possess a significant physical footprint or are primarily designed for ambulatory patients, which precludes their safe and practical use with critically ill, deeply sedated patients in the confined and equipment-dense environment of an ICU. Additionally, although commercial devices must never distort the other equipment, and therefore must comply with Electromagnetic Compatibility (EMC) limits established in EN 60601-1-2 standard [9], this topic is rarely addressed in detail in the PET instrumentation literature.

The PETITION (PET for Intensive Care units and Innovative protON therapy) project was initiated to address this fundamental gap by developing two dedicated mobile brain PET scanners with a target spatial resolution of better than 4 mm in the center of the Field of View (FOV) for an activity of up to 50 MBq in the FOV. The primary aim of this work is to present the design and development of the system, together with the results of the initial performance evaluations and electromagnetic compatibility (EMC) test results.

To maximize the synergies between the two sub-projects, we developed a modular and highly integrated detector concept. The electronics is based on previous PET detector designs from our group [10,11] and has been improved to reduce Electromagnetic Interference (EMI) and match the required form factors. The focus of our development was the small form factor and mobility. Therefore we decided against a time-of-flight design in order to decrease power consumption and consequently cooling requirements. However, we still want to use a narrow coincidence window, determined by the size of the Field of View and not by our timing resolution, in order to keep random coincidences under control.

In this work, we present the hardware developed for both systems in detail, along with initial performance evaluations and EMC test results. We provide a description of the custom hardware, electronics, and firmware architecture, with a particular focus on the design choices and empirical validation for achieving compliance with the EN 60601-1-2 standard for electromagnetic emissions, which is rarely found in the existing literature.

The remainder of this paper is structured as follows: Section 2 details the materials and system design, including the modular concept and electronic components. Section 3 describes the methodologies employed for both electromagnetic emissions testing and Positron Emission Tomography imaging performance evaluation. Section 4 presents the results obtained from these measurements. Section 5 provides a comprehensive discussion of our findings, their implications, and the current limitations. Finally, Section 6 offers a concise summary of our conclusions and outlines future work.

## 2. Materials

### 2.1. System Design

**Figure 1:**
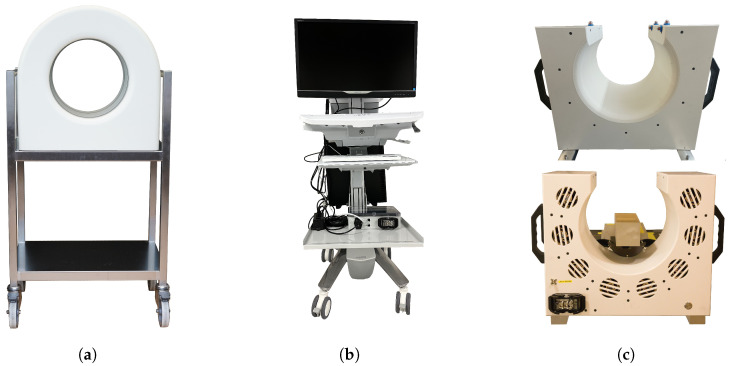
Components of the PET system: (**a**) Front view of the ICU scanner-trolley. (**b**) Operator’s trolley, containing the computer for image reconstruction and the 24 V power supply for the scanner. (**c**) PBT scanner, featuring an opening for proton irradiation. The top image shows the scanner viewed from the front, and the bottom image shows it viewed from the back.

To enable the required mobility and small footprint, both PET systems comprise two parts: the PET detector itself on one trolley and a computer for system control on a second trolley. The scanner trolley (Figure 1a) holds the PET scanner and can be pushed and locked in place at the end of the patient’s bed in the ICU or the patient couch in the PBT Gantry. The operator’s trolley (Figure 1b) contains the power supply and the Data acquisition (DAQ) computer for system control, data processing, and image reconstruction. It remains outside the ICU room or beam area to minimize the footprint of the PET system. Both trolleys are connected by a flexible 15 m cable harness that brings the 24 V DC operating power to the scanner and the data communication through two 10 Gbps Ethernet cables. The harness housing protects the cables from water and dust according to IP68, and a robust connector prevents its unintentional unplugging.

The PBT PET system (Figure 1c) is mounted directly to the patient couch in the Gantry of the proton beam facility. The same cable connection is used to allow for undisturbed movement of the patient couch. The operator’s trolley is similarly placed away from the beam line.

### 2.2. PET Scanner

The PET scanner is highly integrated and contains all the electronics required for the detection of 511 keV gamma rays and digitization of the information. Figure 1a shows the front view of the ICU scanner as seen from the patient side. Both scanners have a bore diameter of 300 mm and an axial field of view of 179 mm in length. The rear part of the scanner holds the connector to the cable harness and exhibits small slots for air outlet. These slots are visible in the PBT scanner shown in Figure 1c. The PBT scanner features an additional opening of 224 mm to allow to perform proton irradiations to the sample in the scanner.

**Figure 2:**
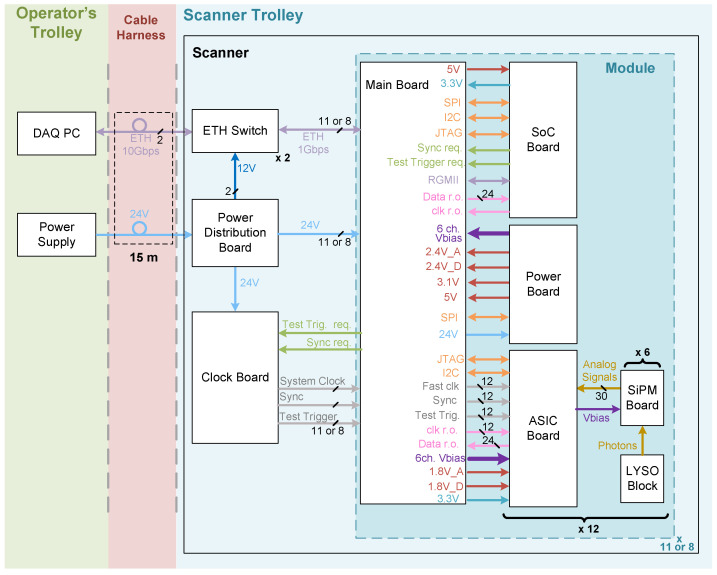
Simplified block diagram of the PET system.

Figure 2 shows a simplified block diagram of the developed system, which is detailed in the following sections. The scanners are composed of several modules arranged to form a circle (in the case of the ICU scanner) or a C shape (in the case of the PBT scanner). All the modules are identically built and function independently. There are two main differences between the ICU and PBT scanner. First, the number of modules used to achieve their geometries, with 11 and 8 modules, respectively. Second, the PBT scanner has a coaxial input connected to the trigger signal of the beam line (which is not installed in the ICU system), enabling us to timestamp the moments when the beam is on or off. This is used to decrease the data rates during beam on, reducing counts from the prompt gammas.

**Figure 3:**
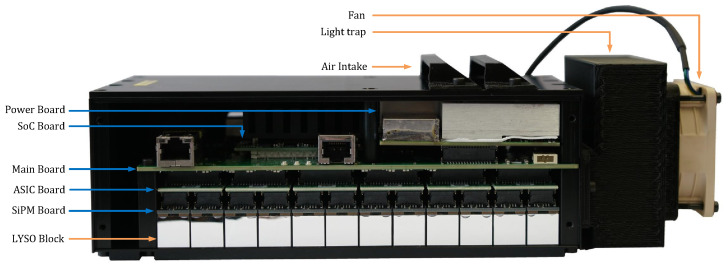
Side view of a module without the lateral lid, showing the electronics. Seventy-two LYSO blocks are arranged in a 12×6 array. Next comes a layer of 6×2 ASIC Boards, each reading out the Silicon PhotoMultipliers (SiPMs) of 6 crystal blocks. Above the Application-specific integrated circuit (ASIC) boards comes the Main Board handling all digital data and interfaces. Finally, the SoC module and the Power Board are on top.

Each module contains scintillation crystals, photodetectors, and electronics for detecting gamma rays and outputs information regarding the energy and time at which these gamma rays were detected. Figure 3 displays a side view of the module without its lateral lid, with the bottom oriented towards the patient’s head. The electronics are housed in a 216 mm × 95 mm × 75 mm metallic black box, which prevents any external light leakage and helps reduce the EMI emitted to the exterior. Each module includes the following:

- A custom Printed Circuit Board (PCB), referred to as the Main Board (see Section 2.3.1), located at the center and responsible for holding and connecting all other boards within the module.
- A commercial System on Chip (SoC) module (Enclustra ZX1, Enclustra, Zurich, Switzerland, see Section 2.3.2) that processes the digital data.
- A custom PCB named Power Board (see Section 2.3.4), which generates low-voltage rails for the other boards from a single 24 V input voltage and provides the bias voltage for the photodetectors.
- Twelve custom PCBs, called ASIC Boards (see Section 2.3.5), organized in a 6×2 array. Each board is equipped with a Position-Energy-Timing Application (PETA8) readout chip, developed by the University of Heidelberg, that digitizes the analog signals from the photodetectors (Hamamatsu S14160-4050HS [12]).
- Seventy-two custom PCBs, called SiPM boards (see Section 2.3.6), each containing five photodetectors.
- Seventy-two scintillation crystal blocks composed of Lutetium-yttrium oxyorthosilicate (LYSO) material. The bottom cover contains machined metal strips to hold the crystal blocks precisely in place.
- A commercial-grade fan (Noctua NF-A6x25, Noctua, Vienna, Austria) coupled with a custom-designed light trap.

Both PET systems are equipped with shared components at the base of the scanner, which include the following:

- A Clock Board, detailed in Section 2.3.7,which provides a uniform 25 MHz system clock and synchronization signals to all modules.
- Two commercial Ethernet (ETH) switches (Netgear GS110MX, Netgear, San Jose, CA, USA) which interconnect the modules and the DAQ computer located at the operator’s trolley.
- A power distribution board (see Section 2.3.8) that distributes the 24 V from the power supply at the operator’s trolley to all the electronics within the PET scanner.

### 2.3. Electronics

The pivotal electronic components of our PET system are illustrated in Figure 2 and detailed in this section.

#### 2.3.1. Main Board

The Main Board is a custom PCB located at the center of a module with outer dimensions of 196 mm × 92 mm. It is a 14-layer PCB with several ground and power layers to improve signal integrity and reduce noise emissions of fast switching signals. The 12 ASIC Boards, the SoC module, and the Power Board of a module plug directly into it. It connects directly to the Clock Board (see Section 2.3.7) and via an Ethernet switch to the DAQ computer.

The Main Board routes 24 serial links from the ASIC Boards to the SoC module board (Section 2.3.2) through Low Voltage Differential Signaling (LVDS) differential pairs. Communication with the DAQ computer is achieved via Gigabit Ethernet over copper twisted pair (1000BASE-T). For this purpose, the Main Board hosts a RJ45 connector with magnetics (Bel 0826-1X1T-43-F, Bel Magnetic Solutions, West Orange, NJ, USA) [13] and the Ethernet PHY transceiver Alaska 88E1518 from Marvell Technology, Marvell Technology, Santa Clara, CA, USA [14].

The timing resolution of the PET system is directly impacted by the quality of the clocks used to sample the detector signals. To ensure an ultra-low jitter clock for the high-speed PETA8 ASICs, a dedicated jitter-attenuating clock multiplier chip was required. The Skyworks Si5342, Skyworks Solutions, Irvine, CA, USA [15] was selected because of its exceptional jitter performance, providing a root mean square (RMS) jitter of less than 100 fs on the output clocks. The Si5342 takes the 25 MHz system clock from the Clock Board and generates the required 625 MHz LVDS clock for the PETA8 ASICs and a clock four times slower (156.25 MHz) fed into the SoC module board using a clock-capable pin. To distribute the high-frequency system clock signal across multiple PETA8 ASICs without introducing significant additional jitter, a high-performance clock buffer was essential. The Main Board includes low additive jitter LVDS fanout buffers (CDCLVD1216) from Texas Instruments [16] (low-additive jitter of only 150 fs RMS) to deliver the following signals to all 12 ASIC Boards in a module:

- Sync and Test Trigger signals from the Clock Board.
- The readout clock from the SoC module board.
- The 625 MHz clock signal generated at the Main Board from the 25 MHz system clock.

Additionally, the Main Board includes several slower communication interfaces.

- Four Joint Test Action Group (JTAG) chains connecting three ASIC Boards each to the SoC module for PETA8 configuration.
- Three Inter-Integrated Circuit (I2C) chains for all LTC2991, Analog Devices, Wilmington, MA, USA [17] chips and an EEPROM on the SoC module, with the SoC module acting as the master.
- One Serial Peripheral Interface (SPI) with two slaves, connecting the SoC module to the Power Board for voltage control and monitoring (see Section 2.3.4).

Power regulation on the Main Board is managed using nine TPS7A8400 Low Dropouts (LDOs) from Texas Instruments, Dallas, TX, USA, which regulate voltages from the Power Board closer to the load to improve power integrity. It also routes all six bias voltage channels from the Power Board to the ASIC Boards. Each channel is used to bias two ASIC Boards, thus serving a total of 60 SiPMs. These SiPMs are specifically selected to ensure their breakdown voltage remains within 100 mV.

Temperature monitoring on the Main Board is conducted using an LTC2991 sensor at five strategic locations. One Main Board, identified as the ’master’ but identical to the others, has an output connected to the Clock Board to generate the synchronization signals, while remaining unconnected in the other Main Boards.

#### 2.3.2. SoC Module

We use the Enclustra ZX1 commercial SoC Module, which contains a AMD Zynq-7030 System on Chip [18,19] (Enclustra, Zurich, Switzerland). The AMD Zynq-7030 SoC was selected for its speed and combination of a programmable logic (Programmable logic (PL)) side for high-speed data processing and a processing system (Processing system (PS)) for slower interfaces, a crucial feature for managing the high data rates from the PETA8 ASICs while maintaining system control. The PL consists of a Series 7 Kintex Field Programmable Gate Array (FPGA) [20,21] (AMD, San Jose, CA, USA) and the PS of an ARM-based processor. From all available components of the module, we use the following:

- Clock generation for the PS and PL.
- EEPROM with an available MAC address (I2C communication to PS).
- QSPI flash memory for booting.
- Power management to operate from a single 5 V input voltage.

The PL is responsible of all the fast interfaces (Ethernet link and 24 × 250 Mbps serial input from the PETA8s) and for creating the corresponding data frames. The PS is used to control slower interfaces like I2C and SPI, thus simplifying the PL side.

#### 2.3.3. Firmware

**Figure 4:**
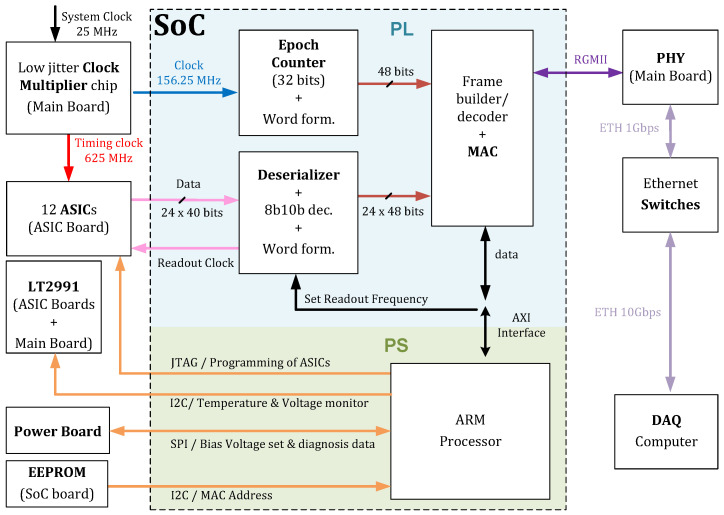
Firmware blocks in the SoC and its connections in the detector module.

All the modules use the exact same firmware. A simplified block diagram of the firmware is shown in Figure 4. We implemented an Ethernet Media Access Controller (MAC) in the PL side of the SoC, which communicates via Reduced Gigabit Media Independent Interface (RGMII) to the PHY transceiver in the Main Board. Each module has a unique MAC address stored in the EEPROM of the SoC board, retrievable by the PS side using I2C. In the DAQ computer, a configuration file maps each module (and its MAC address) to its position in the scanner, allowing the DAQ computer to identify the source of any data received from the Ethernet switches and to send specific instructions to individual modules.

The readout clock is generated by the PL side, buffered by the Main Board, and transmitted to all 12 ASICs. The 24 serial data lines from the 12 ASICs are received at the SoC. A deserializer block in the PL transforms the serial data into a parallel 10 bits, subsequently decoded by the 8b10b decoding block. The ASIC generates 5 consecutive bytes for every hit, used to form a 48-bit word-in-word formation block by regrouping them and attaching information on which of the 24 serial data lines it belongs to. The readout speed can be adjusted from 251 MHz to 266 MHz in 1 MHz steps by the DAQ software, which alters the readout clock frequency and the frequencies of all other clocks required by the deserializer and subsequent blocks, allowing each module to be read at slightly different frequencies.

The PL contains a 32-bit counter, running at 625 MHz/4096 in parallel with the 625 MHz counters in the ASIC. This additional counter is used to detect overflows of the ASIC counters. Several blocks of readout data and epoch counter data are combined in an Ethernet frame and sent to the DAQ computer (ETH Data frame).

The PS side is responsible for configuring each individual PETA8 ASIC as dictated by the DAQ computer using four JTAG chains.

The PS side creates a diagnosis Ethernet frame and sends it every second to the DAQ computer. The most important information contained inside the diagnosis frame is

- Temperature of all ASIC Boards.
- Temperature of 36 SiPM Boards.
- Temperature of 5 points in the Main Board.
- Temperature of the Power Board.
- Voltage value of low voltage rails generated at the Power Board.
- Bias voltage value of one channel.
- Total bias current.
- Fan speed and total input current.

The temperature data from the LTC2991 chip is obtained using I2C communication, while the various voltage values and temperature of the Power Board are read using SPI communication. The bias voltage for each individual channel can be set via SPI and is controlled by the DAQ software. The bias voltage and temperature information are utilized to compensate for changes in the SiPM’s breakdown voltage due to temperature variations, aiming to maintain a constant gain of the SiPMs.

The synchronization and test trigger signals (not shown in the figure) are generated by the PL of the “master board” after receiving a DAQ instruction, and sent to the Clock Board (Section 2.3.7), which buffers and sends them to all the modules.

#### 2.3.4. Power Board

The Power Board is a custom PCB measuring 80 mm × 92 mm and plugs directly into the Main Board. Its primary functions are to generate the bias voltage for the SiPMs and the low-voltage rails required by the Main Board. These low-voltage rails are generated using four step-down Direct Current to Direct Current (DC-DC) converters, whose design has been optimized to reduce EMI following the design recommendations in [22,23]. The DC-DC converter voltages are the following:

- 5 V for powering the SoC module.
- 3.1 V, which is subsequently regulated to 2.5 V on the Main Board for powering the fanout chips.
- 2 × 2.4 V, which are subsequently regulated to 1.8 V on the Main Board for powering the analog and digital sections of the PETA8 ASIC, respectively.

Voltage regulation on the Main Board is performed close to the load using LDO voltage regulators, with decoupling capacitors as close as possible to the ICs.

The ability to independently and precisely adjust the bias voltage of the detector modules is critical for fine-tuning the gain and performance of the Silicon Photomultipliers (SiPMs). Adjustable bias voltages are generated using a 14-bit Digital-to-Analog Converter (DAC) (AD5648, Analog Devices, Wilmington, NC, USA) [24] coupled with multiple power amplifiers (LTC6090, Analog Devices, Wilmington, NC, USA) [25] featuring negative feedback. There are seven such circuits, each utilizing one of the eight channels of the DAC to set a voltage at the input of the power amplifier. This setup allows for the generation of seven independent bias voltage values, adjustable in 3 mV steps. The input voltage for the power amplifier, derived from an inverter DC-DC (LT8361, Analog Devices, Wilmington, NC, USA [26]), generates −48 V from a 24 V input. The bias voltage is adjustable remotely from the DAQ computer via an Ethernet command, which prompts the SoC module to communicate with the DAC using SPI.

Furthermore, a 12-bit Analog-to-Digital Converter (ADC) monitors the output voltages from the DC-DC converters, one bias channel, the total bias current of all channels, and the board’s temperature.

To minimize EMI emissions, the Power Board’s layout was meticulously designed, with all DC-DC converters shielded by 18 μm copper plates, soldered to the ground plane of the PCB and effectively functioning as Faraday cages [22,23].

#### 2.3.5. ASIC Board

The ASIC Board is a custom PCB that connects to six SiPM boards on one side and to the Main Board on the other. It has a total dimension of 43 mm × 28.5 mm. The front-end electronics for each detector module were designed around the PETA8 ASIC, a custom readout chip developed at the University of Heidelberg. This ASIC was selected for its high level of integration and performance, which are critical for the mobile PET scanner’s design goals. Specifically, the PETA8 ASIC provides 32 input channels, enabling it to read out a large number of SiPMs per chip, thus reducing the overall component count and system complexity. Each ASIC Board holds a PETA8 which digitizes the analog signals generated by the 30 SiPMs connected to it (two channels remaining unused in our system). The energy information is captured by a Charge-to-Digital Converter (QDC) with nine-bit resolution, and the timestamp of the hit is created using a Time-to-Digital Converter (TDC) with a 50 ps bin width.

The most important connections to each PETA8 ASIC (routed through the ASIC Board) include the following:

- A total of 30 analog inputs (6×5 inputs for the six SiPM Boards).
- Low-jitter 625 MHz LVDS clock as principal reference clock.
- Two LVDS serial link outputs for data transmission.
- LVDS readout clock.
- Sync LVDS signal.
- Test Trigger LVDS signal.
- JTAG interface for configuration.
- Stable 1.8 V rails for the analog and digital circuitry.

The digital data is transmitted via the two serial links using 8b10b encoding at a speed determined by the readout clock. Each channel generates 40 bits of data per hit. The Sync LVDS signal resets the TDC counters and synchronizes all the PETA8s in a PET scanner when asserted simultaneously. The test trigger LVDS signal can trigger all enabled channels simultaneously, even without a detected pulse, useful for debugging and system calibration. Various parameters of the chip and its individual channels can be externally configured using a JTAG interface.

The ASIC Board also includes an LTC2991 temperature monitor to measure the temperature of three out of six SiPM boards attached to the ASIC Board and its own temperature. Additionally, it monitors the voltage on the 1.8 V line used by the ASIC. The LTC2991 is configured and read out using an I2C interface. Therefore, the additional connections needed by the ASIC Board are the following:

- I2C data and clock signals for controlling the LTC2991 temperature monitor.
- 3.3 V rail for powering the LTC2991 chip.

#### 2.3.6. SiPM Board

The SiPM Boards are custom-made and measure 14.15 mm × 14.15 mm. Each board holds five SiPMs and the crystal detector on one side and an NPN transistor for temperature monitoring and a connector for the signals on the other side. To ensure optical isolation and prevent crosstalk, a design consideration was the selection of the PCB’s solder mask color. The solder mask on the SiPM side of the board is white to maximize the reflection of scintillation light, thereby improving light collection efficiency and energy resolution. Conversely, the solder mask on the connector side is black to absorb any stray light, which helps minimize optical noise and ensures the integrity of the data signals. The most important signals to the SiPM board are as follows:

- Five output analog signals, one for each SiPM.
- Bias voltage shared by all the SiPMs.
- NPN temperature signals.

#### 2.3.7. Clock Board

Maintaining precise timing and synchronization across all modules is a critical requirement for achieving high timing resolution and accurate coincidence detection. To meet this need, a dedicated Clock Board was designed. The board’s primary function is to generate and distribute a stable, low-jitter system clock and control signals to all detector modules.

The Clock Board is a custom PCB measuring 196 mm × 70 mm, placed inside the PET scanner. It inputs two signals from one Main Board (sync and test trigger) and outputs three signals (25 MHz system clock, sync and test trigger) to all the modules in the PET scanner. All these signals are transmitted through twisted pairs of an Ethernet cable that plugs in using RJ45 connectors without magnetics.

A 25 MHz system clock is generated using a Clock Oscillator (531FC25M0000DGR from Skywork, Irvine, CA, USA [27]) selected for its ultra-low jitter performance and is fanned out to all the Main Boards using low-additive-jitter LVDS fanout buffers from Texas Instruments (CDCLVD1216). Similarly, the sync and test trigger signals coming from the ‘master’ Main Board are fanned out using two CDCLVD1216 chips. All Ethernet output cables are of the same length to reduce signal skew.

The Clock Board receives 24 V from the Power Distribution Board and internally generates the required 2.5 V rail using an LMZ14203 DC-DC converter [28] and a TPS7A8300 LDO from Texas Instruments, Dallas, TX, USA [29].

#### 2.3.8. Power Distribution Board

Reliable and protected power distribution is a fundamental requirement for any complex electronic system, particularly a medical device operating in a clinical environment. To ensure the stability and safety of the mobile PET scanner, a custom Power Distribution Board measuring 196 mm × 70 mm was designed. It inputs the 24 V power supplied by the Power Box at the operator’s trolley and provides 12 outputs for 24 V and 2 outputs for 12 V. The latter 12 V outputs are generated using two independent LMZ14203 DC-DC converters and used to power the Ethernet switches (Section 2.3.10). System-wide protection against power-related failures was a key design priority. The 24 V input is protected against reverse voltage and voltage spikes using a reverse-bias diode (RURG5060) and a Transient Voltage Suppression (TVS) diode (20KPA26CA). Each output is protected with an independent fuse.

#### 2.3.9. Power Box

The power supply system for the mobile PET scanner was designed with a strong emphasis on electromagnetic compatibility (EMC) and thermal management, which are critical considerations for a medical device intended for clinical environments. The entire assembly, housed in a non-conductive box (Hammond 1554X2GYCL, Hammond Manufacturing, Guelph, ON, Canada), is located on the operator’s trolley and measures 300 × 200 × 90. To ensure compliance with the EN 60601-1-2 medical EMC standards, a commercial Alternating Current to Direct Current (AC-DC) power supply from Meanwell, Fremont, CA, USA (model RPS-500-24) [30] was selected. This power supply provides the required 24 V DC output and serves as the foundation for the system’s power. To further reduce conducted emissions, an additional TDK B84776M, TDK, Chuo City, Japan medical-grade EMC input filter was integrated at the AC input [31]. This design choice is crucial for ensuring the system does not interfere with other sensitive medical equipment. To suppress both AC and DC common-mode noise, which can be a significant source of unwanted signal interference, TDK ZCAT3035-1330 ferrite common-mode chokes [32] were strategically placed on both the AC and DC power lines. This dual-stage filtering approach provides robust noise suppression, protecting both the external power grid from system-generated noise and the scanner’s sensitive electronics from power line interference. Finally, to maintain long-term reliability and prevent component degradation, a Noctua 40 mm 12 V fan was incorporated to enhance thermal management within the power box.

#### 2.3.10. Ethernet Switches

The data from each of the detector modules must be sent to the DAQ computer for processing. To reduce the number of physical connections to the DAQ computer and simplify system cabling, a networked data transmission scheme was implemented using commercial Ethernet switches. Two Netgear GS110MX switches (Netgear, San Jose, CA, USA) were selected for this purpose. This model was chosen because it provides a combination of multiple 1 Gbps Ethernet ports for connecting to the individual detector modules and a high-speed 10 Gbps port for connecting to the DAQ computer via the 15 m long cable.

### 2.4. Block Detector and Photodetectors

A total of 72 block detectors consisting of lutetium–yttrium oxyorthosilicate (LYSO) scintillators per module stop the 511 keV gamma rays. Each block detector is comprised of a 5×5 array of crystal elements, each measuring 2.74 mm × 2.74 mm × 15 mm. The crystals are optically separated by Enhanced Specular Reflector (ESR) foil and have a pitch of 2.81 mm, resulting in a total block outer dimension of 14.14 mm × 14.14 mm × 15 mm. The blocks are arranged in a 6×12 array with a pitch of 15 mm in both directions, thus offering an active area of (89 × 179) mm^2^ per module. A thin plastic grid provides additional optical insulation between the blocks.

Each block is optically coupled to five SiPMs manufactured by Hamamatsu, Shizuoka, Japan (model S14160-4050HS [12]), which are soldered onto the SiPM Board. These solid-state photodetectors, each with a sensitive area of (4 × 4) mm^2^, enable the assignment of events to one of the 25 crystal elements (by the DAQ computer) based on the intensity of the readings from the 5 SiPMs that share one block detector.

### 2.5. Air Cooling

Effective thermal management is a critical design consideration for high-performance electronic systems, as excessive temperatures can degrade component performance and reliability. In this mobile PET scanner, an active air cooling system was implemented for each detector module to dissipate the approximately 25 W of power. The cooling system utilizes a Noctua NF-A6x25 fan (Noctua, Vienna, Austria), powered by 5 V from the Main Board. This fan was selected for its optimal balance of high airflow and low noise, a crucial feature for a medical device that may be operated in a patient-adjacent environment.

To ensure that the cooling system does not compromise the optical integrity of the detector, the air intake and fan are separated by a custom-designed light trap (see Figure 3). This passive component is engineered to allow unobstructed airflow while preventing ambient light from entering the detector module, thereby protecting the highly light-sensitive SiPMs from unwanted optical noise. This design ensures that the cooling system maintains thermal stability without introducing artifacts into the acquired data.

To further enhance thermal transfer, heat sinks were attached to the Power Board and the SoC module. These heat sinks increase the surface area for convective heat dissipation, which significantly reduces the steady-state temperature of these critical components.

### 2.6. DAQ Computer and DAQ Software

We use an Intel Ethernet Converged Network Adapter X550-T2 (Intel, Santa Clara, CA, USA) for the input of the 10 Gbps links. The main task of the DAQ software is to offer a graphical user interface, to control and monitor the detector system, and to perform real-time coincidence sorting. The data processing is performed in a data pipeline with several steps.

1. Events are grouped per detector block and sorted in time.
2. Based on the timestamp, the information is combined to identify the interaction crystal within a block and to apply calibration information specific to each crystal element.
3. The processed data are filtered with a configurable energy window around 511 keV (by default, it is 435 keV to 600 keV).
4. The remaining events are sorted in time.
5. Event pairs are searched that form coincidences (2 ns coincidence window).
6. These are further filtered by applying a cut on the minimal geometric distance between the events.
7. All coincidences, together with timestamps and singles rate information, are stored in a binary list mode file for image reconstruction.

## 3. Methods and Measurements

This section details the methodology and procedures employed for evaluating the electromagnetic emissions of the PET system and assessing the PET imaging performance. Different measurements were executed in the ICU PET system and the results are presented in Section 4.

### 3.1. Electromagnetic Emissions

The PET system is intended for use close to other sensitive equipment. Therefore, its emission levels must be below those dictated by the EN 60601-1-2 standard [9]. The emissions of the PET scanner were measured at the RUAG Test Competence Center in Thun, Switzerland. The results were compared against the limits established for Group 1 Category A equipment. The following tests were performed:

- Conducted disturbances from 150 kHz up to 30 MHz (EN 55011 [33]).
- EN 61000-3-2 Harmonics [34].
- EN 61000-3-3 Flicker [35].
- EM radiation disturbance from 30 MHz up to 1 GHz (EN 55011).
- EM radiation disturbance from 1 GHz up to 6 GHz (EN 55032 [36]).

The measurements are described below.

#### 3.1.1. EM Radiation Disturbance from 30 MHz up to 1 GHz

**Figure 5:**
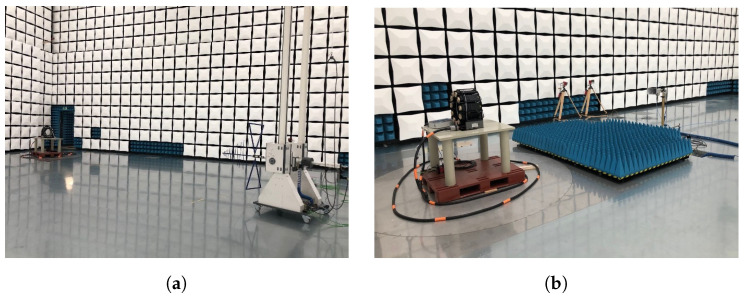
Radiated test setup (**a**) in the 30 MHz to 1 GHz range and (**b**) the 1 GHz to 6 GHz range. (in both pictures, the PET scanner is without the plastic cover, which is not expected to affect the performed measurements).

The measurements were performed in a semi-anechoic chamber with radio-frequency absorbing material on the walls and ceiling, while the floor has a ground plane, according to standards EN 55011 and CISPR 16-2-3 [37]. The maximum radiated interference emission at each frequency in the 30 MHz to 1 GHz range was determined by means of height and polarity scanning of the antenna and rotation of the device under test. The antenna’s height can be varied from 1m to 4m and can be positioned in either horizontal polarity (parallel to the floor) or vertical polarity (perpendicular to the floor). The BiConiLog antenna (model CBL-6111 from Chase) is placed 10m away from the device under test (Figure 5a). The scanner system is placed on a non-conductive table 0.8 m above the ground plane, which can rotate 360 degrees. The antenna is connected to the EMI Receiver model ESW44 from Rohde & Schwarz, configured with a peak detector and a resolution bandwidth of 120 kHz. The resulting radiated disturbance is compared against the applicable quasi-peak limits for Class A Group 1 equipment. If all measured peaks obtained using a peak detector are lower than the quasi-peak limits, the equipment under test passes (as the quasi-peak detector gives a lower reading than the peak detector). If the limit is exceeded, the measurement is repeated at those frequencies using a quasi-peak detector, and the results are compared against the limits.

#### 3.1.2. EM Radiation Disturbance from 1 GHz up to 6 GHz

This test is not required by the standards for our device; nevertheless, it helps us to evaluate the radiated emissions beyond 1 GHz. It is performed in an anechoic chamber, according to standards EN 55032 and CISPR 16-2-3. The maximum radiated interference emission at each frequency in the 1 GHz to 6 GHz range is determined by means of a polarity scan of the antenna and rotation of the device under test. The antenna’s height is 1m and can be positioned in either horizontal or vertical polarity. The Horn antenna (model BBHA 9120D from Schwarzbeck, Mess-Elektronik, Schönau, Germany) is placed 3m away from the device under test. The scanner system is placed on a non-conductive table 0.8m above the ground plane, which can be rotated 360 degrees (Figure 5b). The antenna is connected to the EMI receiver model ESW44 from Rohde & Schwarz, Munich, Germany, configured with peak and average detectors with a resolution bandwidth of 1 MHz. The resulting radiated disturbance must be compared against the applicable peak limits for Class A Group 1 equipment, which correspond to 56(76) dB μVm^−1^ until 3 GHz and 60(80) dB μVm^−1^ at higher frequencies for the average(peak) detector.

#### 3.1.3. Conducted Disturbances, Harmonics, and Flicker

**Figure 6:**
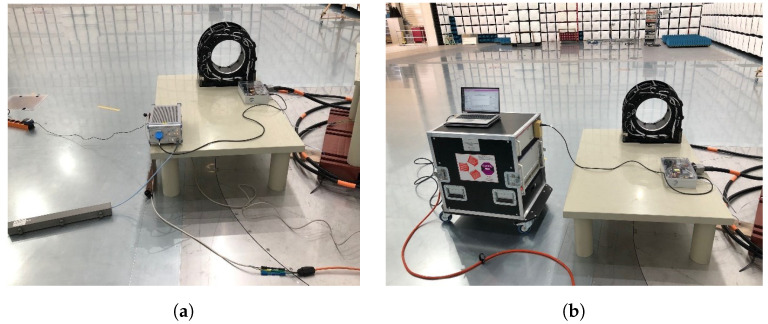
(**a**) Conducted test setup in the 150 kHz to 30 MHz range. (**b**) Harmonics and flicker setup.

The conducted-disturbance test was performed in the same semi-anechoic chamber used in the radiated emissions test (Section 3.1.1). The PET scanner system is placed over a 0.4 m tall non-conductive table, with its AC power cord connected to the mains via an ENV216 Line Impedance Stabilization Network (LISN) from Rohde & Schwarz (Figure 6a). The LISN provides a path for the noise signal in the power lines to be measured by the EMI receiver (model ESW44 from Rohde & Schwarz) and establishes a consistent impedance for the Device Under Test (DUT) in the 150 kHz to 30 MHz range. It also ensures that the measurements accurately reflect the conducted emissions of the DUT alone, free from external influences.

The EMI receiver is configured with a quasi-peak detector and a resolution bandwidth of 9 kHz. The resulting conducted disturbance is compared against the applicable quasi-peak limits for Class A Group 1 equipment, which are 79 dB μV in the 150 kHz to 500 kHz range and 73 dB μV in the remaining 500 kHz to 30 MHz range.

The harmonics test (EN 61000-3-2 standard) is designed to assess the harmonic current emissions of electrical and electronic equipment that draw up to 16 A per phase from the power supply. The objective is to ensure that the equipment does not contribute to excessive distortion of the power line’s voltage waveform, which can adversely affect power quality. The PET scanner system’s power cord is connected to a power supply that mimics a standard network (EM Test DPA 500) and measures the harmonic current. The measurement covers harmonics up to the 40th order of the fundamental frequency (50 Hz). The results are compared against the limits specified in EN 61000-3-2 to determine compliance (Figure 6b).

The flicker test (EN 61000-3-3 standard) evaluates the potential of electronic equipment to cause voltage fluctuations on the power supply that can lead to perceptible light flicker. This test is relevant for equipment with a current rating of less than or equal to 16 A per phase. The test measures the flicker severity produced by the DUT over specific time intervals, utilizing a flickermeter that conforms to the specifications of EN 61000-4-15 [38]. The flickermeter generates the short term flicker (*P*_st_) according to a statistical process over a 10 min observation interval. The long term flicker (*P*_lt_) is calculated over a 2 h observation interval as the cubic average of several *P*_st_ values. Compliance is determined by comparing the measured *P*_st_ and *P*_lt_ values against the thresholds defined in EN 61000-3-3 (1.0 and 0.65, respectively). Moreover, voltage change characteristics in the parameters D_c_, D_max_, and T_max_ are evaluated as defined in EN 61000-3-3 Flicker [35].

### 3.2. PET Imaging

#### 3.2.1. Imaging a Point Source: Timing Resolution and Energy Resolution

To assess the basic performance parameters of our PET system, we conducted data acquisition using a ^22^Na point source, with an activity of 200 kBq, positioned at the center of the FOV.

Before data acquisition for performance assessment, the system underwent an offline calibration procedure once after its assembly.

First, energy calibration was performed. For each individual detector channel (crystal), the measured QDC values from the five associated SiPMs were summed. These summed QDC values were then used to generate “raw spectra” per channel. Afterwards, the photo peak was fitted and a linear calibration constant was obtained based on that value. These unique calibration constants for all crystals are stored in a dedicated configuration file, which is loaded during the scanner’s startup to convert raw QDC values into energy values in keV.

Following energy calibration, timing calibration was also performed on a per-crystal basis. This also utilized data from a point source measurement with a centered source position. The time differences for all crystal pairs with at least 100 recorded coincidences were fed into a minimizer (CERN ROOT’s TMinuit). The objective of this minimization was to assign a unique delay value to each crystal such that the mean time difference of all coincidence pairs, after applying these delays, was as close to zero as possible. These derived delay values were then added to the same calibration file and loaded during system startup to ensure accurate timing measurements.

Using the calibrated data from the ^22^Na point source, we analyzed the coincidence events. Initially, we generated a timing spectrum, indicative of the time difference between pairs of detected events. The system’s timing resolution was quantified by calculating the Full Width at Half Maximum (FWHM) of this distribution. We obtained the maximum by fitting a parabola through the highest bin and its neighbours and interpolated the width at half that value.

Following this, we constructed an energy spectrum from the recorded coincidences. The energy resolution was evaluated by fitting a Gaussian function in the range from 480 keV to 580 keV and reporting the FWHM value of that fit.

#### 3.2.2. Imaging a Hot-Rod Phantom: Spatial Resolution and Maximum Activity

Finally, we employed a hot-rod phantom, which included rods with diameters ranging from 2.4 mm to 1.7 mm (2.4 mm, 2.2 mm, 2.0 mm, 1.9 mm, 1.8 mm and 1.7 mm). The center-to-center distance between adjacent rods was set to twice their respective diameters. This phantom was filled with a 60 MBq ^18^F solution. Prior to imaging, we ensured that the system reached a stable state with no observable systematic package loss. The acquisition commenced at this stabilized activity level—being the maximum activity the PET scanner could handle without data loss—and lasted for 10 min. Data collected during this procedure were then reconstructed using an in-house-developed ordered subset maximization optimization algorithm. The cubic voxels are 0.94 mm × 0.94 mm × 0.94 mm in size and we use 10 subsets, 4 iterations and 1.4 mm Gaussian smoothing after each iteration. The runtime of the reconstruction is about 60 min. The obtained image was then utilized to determine whether the ICU PET system achieved the targeted spatial resolution.

## 4. Results

### 4.1. Electromagnetic Emissions

#### 4.1.1. EM Radiation Disturbance 30 MHz up to 1 GHz

**Figure 7:**
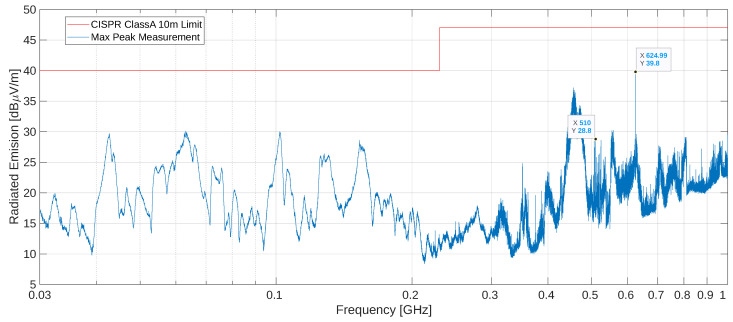
Resulting electromagnetic radiation disturbance in the 30 MHz to 1 GHz range: All peaks obtained using a peak detector are below the quasi-peak limits, thereby indicating compliance with the test.

The resulting radiated emissions of the PET scanner when powered on and fully operational is plotted in blue in Figure 7 (using a peak detector), and the standard limit for a 10 m distance is indicated by the red line (40 dB μVm^−1^ until 230 MHz and 46.5 dB μVm^−1^ for higher frequencies). The radiated emissions stay under the quasi-peak limit across the entire 30 MHz to 1 GHz range by more than 15 dB, thus passing this test. One can recognize harmonics of the 25 MHz system clock and the 125 MHz Ethernet clock, as well as the fundamental of the 625 MHz fast clock for the PETA ASIC, which is the highest peak in the spectrum (39.8 dB μVm^−1^). All modules use a slightly different readout frequency, ranging from 252 MHz to 262 MHz in 1 MHz steps, effectively spreading the spectrum instead of concentrating it at the same frequency. The readout clocks and readout data have spectrum peaks at multiples of those frequencies. An interesting frequency range for investigation is the 500 MHz to 525 MHz, where we can find the second harmonics of the readout clocks.

Table 1 compares the 500 MHz to 525 MHz spectral peaks of Figure 7 (hereafter referred to as the **production setup**) with the peaks from a pre-compliance test using an earlier scanner system’s prototype with all the modules read out at the same frequency of 250 MHz (hereafter referred to as the **prototype setup**). This earlier scanner system’s prototype featured a plastic housing, which we replaced with a conductive housing for the final system (electrically connected to the Main Board PCB’s ground [39]) as one of the strategies to further reduce the emissions. The peak at 525 MHz is not expected to be affected by spreading the readout frequencies of the modules but does show a reduction of 16 dB in the **production setup**, which we attribute to the use of conductive housings for the modules.

In the **prototype setup**, we obtained a peak of 55.3 dB μVm^−1^ at 500 MHz, which surpassed the limits. This peak corresponded to a common harmonic of the 25 MHz system clock, the 125 MHz Ethernet clock, and the module readout clocks and data. Once all modules are read out at slightly different frequencies (**production setup**), the peak at 500 MHz decreases to 21 dB μVm^−1^ (a 34.3 dB reduction), with the power spread across 11 peaks from 504 MHz to 524 MHz, as listed in Table 1.

The average of those 11 peak values is 26.2 dB μVm^−1^, which would account for an equivalent emission of 26.2 + $20\log(\sqrt{11})$ = 36.6 dB μVm^−1^ if the power of the 11 peaks were concentrated at the same frequency of 500 MHz. To compare the total equivalent emissions at 500 MHz for the **production setup** with the 500 MHz emissions measured for the **prototype setup**, we add to the 36.6 dB μVm^−1^ of the 11 peaks the 21 dB μVm^−1^ obtained at 500 MHz and the 16 dB of the previously estimated shield effect, giving a total of 52.8 dB μVm^−1^ if we assume incoherent addition (which uses the root mean square of the fields). That value is close to the measured 55.3 dB μVm^−1^, comfirming that the spreading of the clock frequencies and the proper shielding significantly reduce the peak emissions.

#### 4.1.2. EM Radiation Disturbance: 1 GHz up to 6 GHz

**Figure 8:**
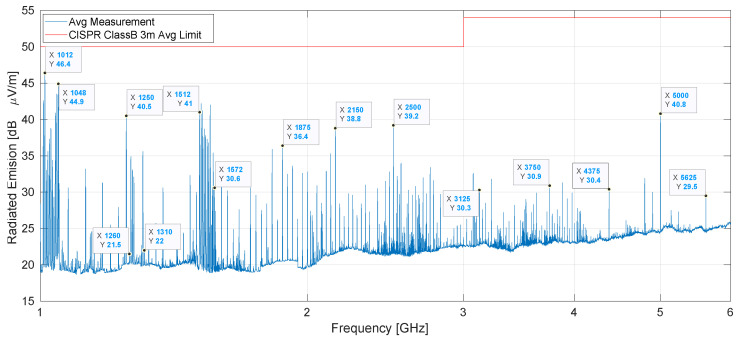
Resulting electromagnetic radiation disturbance in the 1 GHz to 6 GHz range using average detector. Since all peaks are below the limit, the test is passed.

The measurements in the 1 GHz to 6 GHz range were taken using the setup described in Section 3.1.2 and depicted in Figure 5b. The results using an average detector are shown in blue in Figure 8, and the average limits for Class B equipment are in red. All spectrum peaks fall below these limits, which are 6 dB more restrictive than for Class A equipment, thereby passing the test. The largest peak corresponds to the fourth harmonic of the 253 MHz readout clock, being 3.6 dB below the Class B limits and 9.6 dB below the Class A limits. Adjacent to this, the peak at 1048 MHz marks the latest of the fourth harmonics group and the second highest of them. Groups of harmonics of the readout frequencies up to the sixth harmonic (1512 MHz to 1572 MHz) are identifiable. Beyond this frequency, the readout’s contribution to spectrum peaks diminishes significantly, with emissions at least 20 dB below the Class A limits. The strategy of having slightly different readout frequencies contributed positively to test compliance in this frequency range.

Other peaks that can be identified include common harmonics of the 25 MHz system, 125 MHz Ethernet, and 625 MHz fast clocks, labeled as 1250, 1875, 2500, 3125, 3750, 4375, 5000, 5625 MHz. The highest among these, at 1250 MHz (40.5 dB μVm^−1^) and 5000 MHz (40.8 dB μVm^−1^), maintain a margin of more than 15 dB from the Class A limits.

Measurements using a peak detector comply by more than 16 dB with the Class A limits but are not presented.

#### 4.1.3. Conducted Disturbances, Harmonics, and Flicker

The conducted tests were carried out as described in Section 3.1.3 and are depicted in Figure 6a. Both quasi-peak and average detector results have all harmonics under the limits, thus passing the test. Nevertheless, some spectral peaks close to the limit lines in the frequency range of 2.5 MHz to 4.1 MHz are summarized in Table 2. The peak at 2.82 MHz is the largest, with a 5.85(229) dB margin with respect to the quasi-peak (average) limits. All the rest of the peaks have at least a 7 dB margin to their respective limits.

From the harmonic test, we obtained the average and maximum values of the first 40 current harmonics of the PET scanner system. Table 3 lists the harmonics with the highest values, disregarding those less than 0.6% of the input current or less than 5 mA, whichever is greater. The effective value $I_{\mathrm{eff}}$ of the fundamental corresponds to 1.194 A (average) and 1.196 A (maximum). All applicable harmonics (3, 5, 7, 9, 11, 13, 35, and 37) have passed both average and maximum conditions, thus passing the test. There is a considerable margin with respect to the limits, which can be observed in the “percentages of the limits” columns (well below 100%).

The PET scanner system has passed the flicker test during an observation time of 12×10min, with *P*_lt_ and maximum *P*_st_ values 20 times lower than the limit (shown in Table 4). Both *P*_lt_ and maximum *P*_st_ have the same value, which indicates that the PET scanner system behaved in a relatively constant fashion during the whole test. Additionally, the directly measured flicker parameters (maximum D_c_, maximum D_max_ and maximum T_max_) are well below the limits set by the IEC 61000-3-3 standard, as shown in Table 4.

### 4.2. PET Imaging

#### 4.2.1. Imaging a Point Source: Timing Resolution and Energy Resolution

Using a ^22^Na point source placed at the center of the field of view (see Section 3.2), we generated a timing spectrum and energy spectrum, which allow us to calculate two key performance metrics for our PET system: timing resolution and energy resolution.

**Figure 9:**
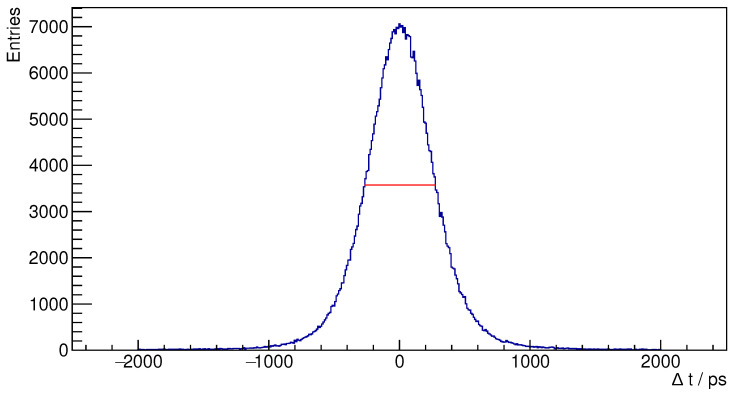
Timing spectrum for the ^22^Na source.

The timing spectrum exhibits a Gaussian shape centered at zero (Figure 9), as anticipated. The system’s timing resolution, represented by the FWHM of this distribution, is 548 ps.

**Figure 10:**
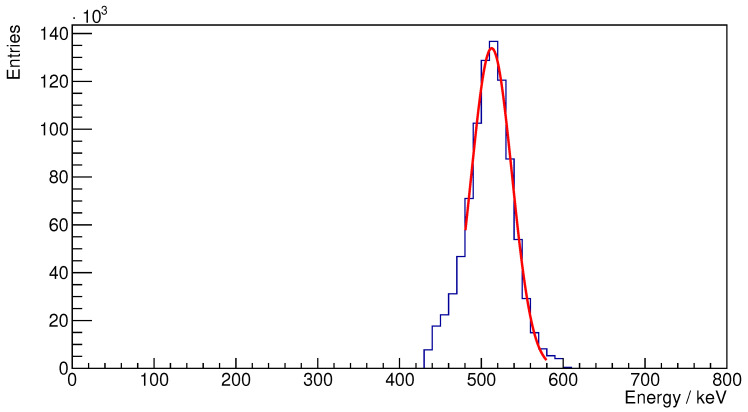
Energy spectrum for the ^22^Na source.

Subsequently, Figure 10 illustrates the energy spectrum derived from the observed coincidences alongside the Gaussian fit applied to these data. The fitting process identified a peak energy of 512.6 keV, indicating successful calibration of the system. The energy resolution, calculated using the formula $\frac{\mathrm{FWHM}}{\mathrm{Peak}\;\mathrm{Energy}}\times 100\%$ is determined to be 11.4%. This metric is in line with anticipated performance, considering the limited coverage of the SiPM readout in relation to the crystal surface (5 SiPMs with 4 × 4 mm^2^ sensitive area on a 14.1 mm × 14.1 mm crystal surface, approximately 40% coverage). This design choice reflects a deliberate compromise aimed at minimizing the overall size and power requirements of our system.

**Figure 11:**
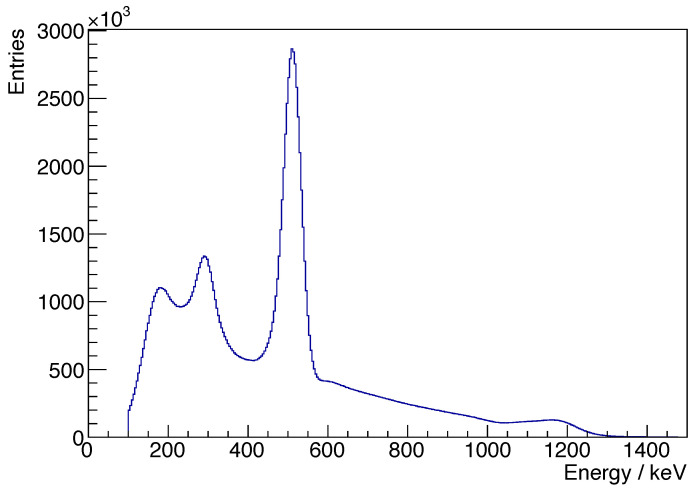
Singles energy spectrum for a ^22^Na source.

The singles energy spectrum acquired from the ^22^Na source is presented in Figure 11. This spectrum clearly displays the 511 keV annihilation photopeak, as well as the peaks for the intrinsic radiation and the prompt gamma peak of the ^22^Na source.

#### 4.2.2. Imaging a Hot-Rod Phantom: Spatial Resolution and Maximum Activity

For image reconstruction, an in-house-developed ordered subset maximization optimization algorithm is used. The cubic voxels are 0.94 × 0.94 × 0.94 mm^3^ in size and we use 10 subsets, four iterations and 1.4 mm Gaussian smoothing after each iteration. The runtime of the reconstruction is about 60 min.

**Figure 12:**
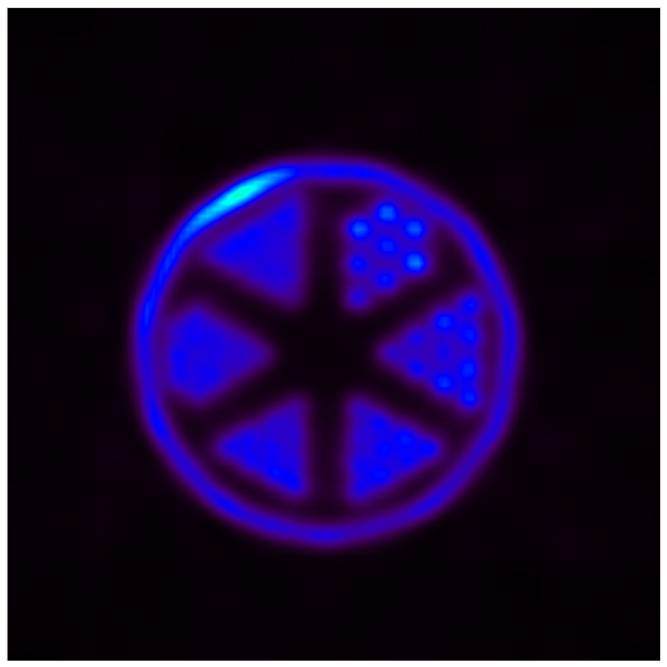
Reconstructed image of a hot-rod phantom (with diameters of 2.4 mm, 2.2 mm, 2.0 mm, 1.9 mm, 1.8 mm and 1.7 mm), which shows that a 2.2 mm spacial resolution can be achieved with our PET scanner.

Above 54 MBq, we experienced data loss due to bandwdith limitations (1 Gbps link of the modules), surpassing our initial target of 50 MBq. The reconstructed image of the hot-rod phantom (Figure 12) closely resembles the actual phantom, indicating the effectiveness of our Brain PET scanner. The image allows us to approximate the system’s spatial resolution based on the discernibility of the rods in the phantom. In the shown slice one can clearly distinguishe the 2.4 mm and 2.2 mm rods. In the 2.0 mm and 1.9 mm rods is some structure visible, while the 1.8 mm and 1.7 mm rods cannot be resolved.

Therefore, we conclude that we can resolve all structures up to 2.2 mm size.

## 5. Discussion

The development and evaluation of two mobile brain PET scanners designed for ICU and PBT applications are presented. Our findings demonstrate the technical feasibility and potential clinical utility of these systems, with specific attention to electromagnetic compatibility for safe use in medical environments.

All tests performed on the ICU PET scanner—conducted emissions, harmonics, flicker, and radiated emissions—complied with the EN 60601-1-2 standard. Radiated emissions, arguably the most challenging to pass, complied with peaks 15 dB below the regulatory limits across the 30 MHz to 1 GHz frequency range, showing the effectiveness of our EMI-oriented design and mitigation strategies. These measures suggest that the scanner can be used in the sensitive electronic environments of ICU and PBT settings without interfering with other medical devices.

Operational testing with a ^22^Na source and a hot-rod phantom revealed an energy resolution of 11.4%, alongside a qualitative spatial resolution approximated at around 2.2 mm (comparable to the PET systems reviewed in [8]). The maximum time difference for the photon pairs in our field of view is about 1000 ps, which was our design limit. We achieved 548 ps of time resolution which is well within this limit but worse then the best time-of-flight systems available [7].

These metrics indicate the scanners’ capability to produce high-quality images, which is essential for accurate diagnosis and treatment monitoring in brain imaging applications.

While our results are promising, we acknowledge certain limitations, such as the preliminary nature of the performance evaluation, the qualitative assessment of the scanner’s spatial resolution, and the need for extensive clinical validation. We also experienced data loss due to bandwidth limitations (the 1 Gbps link of the modules) for activities exceeding 54 MBq. Although this surpasses our initial target of 50 MBq, this limitation should be considered for higher dose applications.

In conclusion, our study offers insights into the design and performance of mobile brain PET scanners. The operational performance expected from our design choices and the scanner’s noise emission levels allow us to use them near other medical equipment. The ICU PET scanner system will be used soon in clinical trials for further validation and the PBT scanner system is being tested with phantoms at the Paul Scherrer Institute (PSI) in Switzerland to validate its use as part of proton therapy treatment.

## 6. Conclusions

This study successfully presents the design, development, and initial performance evaluation of two novel mobile brain Positron Emission Tomography (PET) scanners under the PETITION project, specifically tailored for Intensive Care Unit (ICU) and Proton Beam Therapy (PBT) applications. Our work directly addresses a gap in medical imaging, providing a versatile solution where commercially available systems are absent and existing experimental approaches fall short due to limitations in aperture for PBT or big footprint.

One contribution of this work is the reporting of EMC emission tests. All emission tests performed on the ICU PET scanner, including conducted emissions, harmonics, flicker, and radiated emissions, successfully complied with the EN 60601-1-2 standard. This was achieved through design choices and the implementation of effective Electromagnetic Interference (EMI) mitigation strategies, such as the use of conductive housings, shielded DC-DC converters, and the strategic spreading of readout frequencies.

While comprehensive immunity testing was not conducted, the device was operated in different real environments and exposed to uncontrolled EMI emissions from surrounding electronics without any observed performance degradation. As design principles for reducing emissions often enhance a system’s resilience to external EMI, these practical observations are encouraging. Nonetheless, without formal measurements, we conclude that our device is in partial compliance with the EN 60601-1-2 standard. Full immunity testing remains a subject for future work.

Furthermore, the preliminary performance evaluations confirm the system’s capability to deliver high-quality imaging. Operational testing revealed a timing resolution of 548 ps, an energy resolution of 11.4%, and a qualitative spatial resolution of approximately 2.2 mm.

These metrics meet our initial targets for image quality, essential for accurate diagnosis and treatment monitoring in brain imaging applications. The modular and highly integrated detector concept, allowing for the flexible construction of both circular ICU and C-shaped PBT scanners from largely identical components, underscores the versatility and resource efficiency of our approach, fulfilling a core objective of the PETITION project.

The ICU PET scanner system is under further assessment in clinical trials, and the PBT scanner system continues phantom testing at the Paul Scherrer Institute in Switzerland, for their eventual routine clinical use.

## Figures and Tables

**Table 1:** Comparison of radiated emissions between having a single readout frequency for all the modules (**prototype setup**) and having a slightly different frequency for each of them (**production setup**). The limit at this frequency range is 46.5 dB μVm^−1^.

Freq.	Production Setup	Prototype Setup
**[MHz]**	**[dB μVm^−1^]**	**[dB μVm^−1^]**
500	21.0	55.3
504	22.8	-
506	28.5	-
508	27.4	-
510	28.8	-
512	25.8	-
514	23.1	-
516	24.9	-
518	26.2	-
520	26.4	-
522	23.8	-
524	28.6	-
525	19.4	35.4

**Table 2:** Critical frequencies for the conducted tests in the 150 kHz to 30 MHz interval. For the frequency range of interest (2.5 MHz to 4.1 MHz), the limits are 73 dB μV and 60 dB μV for quasi-peak and average, respectively.

Freq. MHz	QPeak dB μV	Margin dB	CAvg dB μV	Margin Avg dB
2.55	–	–	47.29	12.71
2.64	–	–	51.69	8.31
2.73	–	–	52.36	7.64
2.82	67.15	5.85	57.71	2.29
2.91	–	–	51.25	8.75
3.00	–	–	49.79	10.21
3.18	–	–	48.29	11.71
3.82	–	–	51.72	8.28
3.87	64.52	8.48	–	–
3.91	–	–	51.7	8.30
4.09	–	–	48.13	11.87

**Table 3:** Current test result: average and maximum harmonic current results.

	Average				Maximum			
Hn	$I_{\mathrm{eff}}$ [A]	% of Limit	Limit [A]	Result	$I_{\mathrm{eff}}$ [A]	% of Limit	Limit [A]	Result
1	1.194				1.196			
2	0.007	0.616	1.080	n/a	0.008	0.490	1.620	PASS
3	0.080	3.489	2.300	PASS	0.081	2.348	3.450	PASS
5	0.048	4.189	1.140	PASS	0.049	2.840	1.710	PASS
7	0.016	2.134	0.770	PASS	0.017	1.469	1.155	PASS
9	0.013	3.261	0.400	PASS	0.014	2.256	0.600	PASS
11	0.016	4.834	0.330	PASS	0.016	3.329	0.495	PASS
13	0.008	3.604	0.210	PASS	0.008	2.559	0.315	PASS
35	0.007	10.852	0.064	n/a	0.008	7.898	0.096	PASS
37	0.007	11.179	0.061	n/a	0.007	8.221	0.091	PASS

**Table 4:** Flicker test summary.

	*P*_lt_	Max *P*_st_	Max D_c_ [%]	Max D_max_ [%]	Max T_max_ [s]
Measurement:	0.028	0.028	0	<0.2	0
Limits:	0.65	1	3.3	4	0.5
Results:	PASS	PASS	PASS	PASS	PASS

## Data Availability

The original contributions presented in this study are included in the article. Further inquiries can be directed to the corresponding author.

## Abbreviations

*P*_lt_	Long-Term Flicker
$P_{\mathrm{st}}$	Short-Term Flicker
AC-DC	Alternating Current to Direct Current
ADC	Analog-to-Digital Converter
ASIC	Application-Specific Integrated Circuit
CT	Computed Tomography
DAC	Digital-to-Analog Converter
DAQ	Data Acquisition
DC-DC	Direct Current to Direct Current 0
DUT	Device Under Test
EMC	Electromagnetic Compatibility
EMI	Electromagnetic Interference
ETH	Ethernet
FOV	Field of View
FPGA	Field Programmable Gate Array
FWHM	Full Width at Half Maximum
I2C	Inter-Integrated Circuit
ICU	Intensive Care Unit
JTAG	Joint Test Action Group
LDO	Low Dropout
LISN	Line Impedance Stabilization Network
LVDS	Low Voltage Differential Signaling
LYSO	Lutetium–Yttrium Oxyorthosilicate
MAC	Media Access Controller
MRI	Magnetic Resonance Imaging
PBT	Proton Beam Therapy
PCB	Printed Circuit Board
PET	Positron Emission Tomography
PETA8	Position–Energy–Timing Application
PETITION	PET for Intensive Care Units and Innovative Proton Therapy
PL	Programmable Logic 6
PS	Processing System
QDC	Charge-to-Digital Converter
RGMII	Reduced Gigabit Media Independent Interface
SiPM	Silicon PhotoMultiplier
SoC	System on Chip
SPI	Serial Peripheral Interface
TDC	Time-to-Digital Converter
TVS	Transient Voltage Suppression
